# The Replication Fork: Understanding the Eukaryotic Replication Machinery and the Challenges to Genome Duplication

**DOI:** 10.3390/genes4010001

**Published:** 2013-01-29

**Authors:** Adam R. Leman, Eishi Noguchi

**Affiliations:** Department of Biochemistry and Molecular Biology, Drexel University College of Medicine, Philadelphia, PA 19102, USA

**Keywords:** DNA replication, replisome, replication fork, genome stability, checkpoint, fork barriers, difficult-to-replicate sites

## Abstract

Eukaryotic cells must accurately and efficiently duplicate their genomes during each round of the cell cycle. Multiple linear chromosomes, an abundance of regulatory elements, and chromosome packaging are all challenges that the eukaryotic DNA replication machinery must successfully overcome. The replication machinery, the “replisome” complex, is composed of many specialized proteins with functions in supporting replication by DNA polymerases. Efficient replisome progression relies on tight coordination between the various factors of the replisome. Further, replisome progression must occur on less than ideal templates at various genomic loci. Here, we describe the functions of the major replisome components, as well as some of the obstacles to efficient DNA replication that the replisome confronts. Together, this review summarizes current understanding of the vastly complicated task of replicating eukaryotic DNA.

## 1. Introduction: Basics of DNA Replication

DNA replication, at its most fundamental, is the action of DNA polymerases synthesizing a DNA strand complementary to the original template strand. To synthesize DNA, the double-stranded DNA is unwound by DNA helicases ahead of polymerases, forming a replication fork containing two single-stranded templates. Replication processes permit the copying of a single DNA double helix into two DNA helices, which are divided into the daughter cells at mitosis. The major enzymatic functions carried out at the replication fork are well conserved from prokaryotes to eukaryotes. In practice, the replication machinery is a massive complex coordinating many proteins all working at the site of replication, forming the “replisome”.

**Figure 1:**
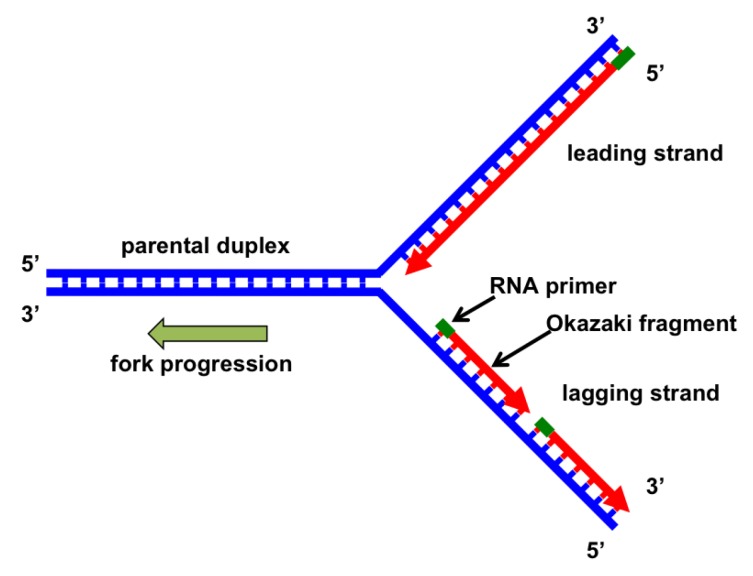
The replication fork. Leading-strand synthesis proceeds continuously in the 5' to 3' direction. Lagging-strand synthesis also occurs in the 5' to 3' direction, but in a discontinuous manner. An RNA/DNA primer (labeled in green) initiates leading-strand synthesis and every Okazaki fragment on the lagging strand.

The replisome is responsible for copying the entirety of genomic DNA in each proliferative cell. This process allows for the high-fidelity passage of hereditary/genetic information from parental cell to daughter cell and is thus essential to all organisms. Much of the cell cycle is built around ensuring that DNA replication occurs without errors. DNA replication is an energetically costly process. In G_1_ phase of the cell cycle, many of the DNA replication regulatory processes are initiated. In eukaryotes, the vast majority of DNA synthesis occurs during S phase of the cell cycle, and the entire genome must be unwound and duplicated to form two daughter copies. During G_2_, any damaged DNA or replication errors are corrected. Finally, one copy of the genomes is segregated to each daughter cell at Mitosis or M phase. These daughter copies each contain one strand from the parental duplex DNA and one nascent antiparallel strand. This mechanism is conserved from prokaryotes to eukaryotes and is known as semiconservative DNA replication [1]. The process of semiconservative replication suggested a geometry for the site of DNA replication, a fork-like DNA structure, where the DNA helix is open, or unwound, exposing unpaired DNA nucleotides for recognition and base pairing for the incorporation of free nucleotides into double-stranded DNA (Figure 1).

The base pairing and chain formation reactions, which form the daughter helix, are catalyzed by DNA polymerases [2,3]. These enzymes move along single-stranded DNA (ssDNA) and allow for the extension of the nascent DNA strand by “reading” the template strand and allowing for incorporation of the proper purine (adenine and guanine) and pyrimidine (thymidine and cytosine) nucleobases. Activated free deoxyribonucleotides exist in the cell as deoxyribonucleotide triphosphates (dNTPs). These free nucleotides are added to an exposed 3'-hydroxyl group on the last incorporated nucleotide. In this reaction, a pyrophosphate is released from the free dNTP, generating energy for the polymerization reaction and exposing the 5' monophosphate, which is then covalently bonded to the 3' oxygen. Additionally, incorrectly inserted nucleotides can be removed and replaced by the correct nucleotides in an energetically favorable reaction. This property is vital to proper proofreading and repair of errors that occur during DNA replication.

**Figure 2:**
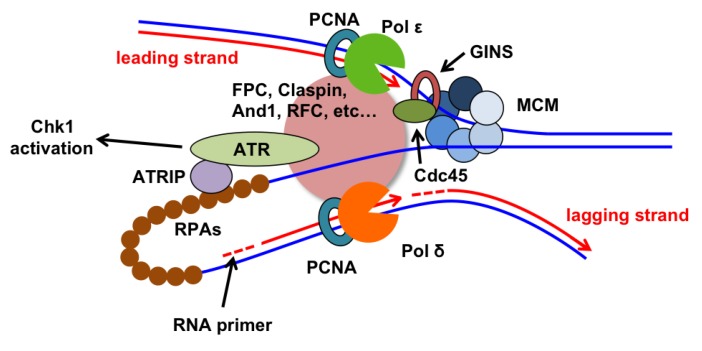
The eukaryotic replisome complex coordinates DNA replication. Replication on the leading and lagging strands is performed by Pol ε and Pol δ, respectively. Many replisome factors (including the FPC [fork protection complex], Claspin, And1, and RFC [the replication factor C clamp loader]) are charged with regulating polymerase functions and coordinating DNA synthesis with unwinding of the template strand by Cdc45-MCM [mini-chromosome maintenance]-GINS [go-ichi-ni-san]. The replisome also associates with checkpoint proteins as DNA replication and genome integrity surveillance mechanisms.

Owing to the antiparallel nature of duplex DNA, DNA replication occurs in opposite directions between the two new strands at the replication fork. However, all DNA polymerases synthesize DNA in the 5' to 3' direction. Consequently, further coordination is required during DNA replication. Two replicative polymerases synthesize DNA in opposing orientations (Figure 1). Polymerase ε (epsilon) synthesizes DNA in a continuous fashion, as it is “pointed” in the same direction as DNA unwinding. This strand is known as the “leading strand.” In contrast, polymerase δ (delta) synthesizes DNA on the opposite template strand in a fragmented, or discontinuous, manner and this strand is termed the “lagging strand” [4]. The discontinuous stretches of DNA replication products on the lagging strand are known as Okazaki fragments and are about 100 to 200 bases in length at eukaryotic replication forks. Owing to the “lagging” nature, the lagging strand generally contains a longer stretch of ssDNA that is coated by single-stranded binding proteins, which stabilizes ssDNA templates by preventing secondary structure formation or other transactions at the exposed ssDNA. In eukaryotes, ssDNA stabilization is maintained by the heterotrimeric complex known as replication protein A (RPA) (Figure 2) [5,6]. Each Okazaki fragment is preceded by an RNA primer, which is displaced by the procession of the next Okazaki fragment during synthesis. In eukaryotic cells, a small amount of the DNA segment immediately upstream of the RNA primer is also displaced, creating a flap structure. This flap is then cleaved by endonucleases (such as Fen1, discussed later). At the replication fork, the gap in DNA after removal of the flap is sealed by DNA ligase I [7,8,9]. Owing to the relatively short nature of the eukaryotic Okazaki fragment, DNA replication synthesis occurring discontinuously on the lagging strand is less efficient and more time consuming than leading-strand synthesis.

## 2. DNA Polymerases and Helicases

The two basic processes of DNA replication are unwinding of the template strand and polymerization of the daughter strands. Therefore, there are two main “workhorse” enzymes in the replisome, the replicative helicase and polymerases. The replicative helicase is responsible for unwinding the parental duplex DNA, exposing two ssDNA templates. The template is then utilized by the replicative polymerases to generate two copies of the parental genome. Recent results have yielded new information on the specific role of each polymerase at the replication fork and while building on our knowledge of the replicative helicase. Understanding the main players in DNA replication allows us to further appreciate their regulation by other replisome factors.

### 2.1. Replicative DNA Polymerases: Further Subdivision of Labor

DNA polymerase function is highly specialized. From viruses through mammals, defined DNA polymerases accomplish replication on specific templates and in narrow localizations. At the eukaryotic replication fork, three distinct replicative polymerase complexes contribute to canonical DNA replication: α, δ, and ε. These three polymerases are essential for viability of the cell [10,11,12,13]. Because DNA polymerases require a primer on which to begin DNA synthesis, first, polymerase α (Pol α) acts as a replicative primase. Pol α is associated with an RNA primase and this complex accomplishes the priming task by synthesizing a primer that contains a short ~10-nucleotide RNA stretch followed by 10 to 20 DNA bases [14,15]. Importantly, this priming action occurs at replication initiation at origins to begin leading-strand synthesis and also at the 5' end of each Okazaki fragment on the lagging strand (Figure 1).

However, Pol α is not able to continue DNA replication. From *in vitro* studies using SV40 T-antigen-dependent replication and reconstituted components, it was observed that DNA replication must be “handed off” to another polymerase to continue synthesis [9,16]. The polymerase switching requires clamp loaders (which will be discussed in detail later in this article) [17]. Initially, it was thought that Pol δ performed leading-strand replication and that Pol α completed each Okazaki fragment on the lagging strand [17,18]. Using mutator polymerase variants and mapping nucleotide misincorporation events, Kunkel and colleagues found that Pol ε and Pol δ mutations lead to mismatched nucleotide incorporation only on the leading and lagging strands, respectively [19,20,21]. Thus, normal DNA replication requires the coordinated actions of three DNA polymerases: Pol α for prime synthesis, Pol ε for leading-strand replication, and the constantly loaded Pol δ for generating Okazaki fragments during lagging-strand synthesis (Figure 2).

### 2.2. Helicases Unwind DNA for Replication

For DNA polymerases to function, the double-stranded helix must be unwound to expose a single-stranded template. This activity is performed by the replicative helicase. In eukaryotes, the replicative helicase is a hexameric complex comprised of the mini-chromosome maintenance proteins (Mcm2-7: Mcm2, Mcm3, Mcm4, Mcm5, Mcm6 and Mcm7). The MCM helicase is an AAA^+^ ATPase, a superfamily of protein complexes that process substrates through a central pore using energy release from ATP hydrolysis [22].

MCM activity is required throughout S phase for DNA replication [23,24]. The MCM proteins are recruited to replication origins (during G_1_ phase and before DNA replication) then redistributed throughout genomic DNA during S phase, indicative of their localization to the replication fork [25]. Although it was known that MCM proteins are required for DNA replication initiation and progression, it was not originally clear what the enzymatic function of the MCM complex could be [26]. In a study using purified MCM homologue from archaea, ATP-driven helicase activity was detected in fractions corresponding to double hexamer forms of the complex [27]. Further, purified complexes of Mcm4/6/7 have ATP-dependent helicase activity *in vitro*, unwinding DNA in the 3' to 5' direction [28,29]. These results, coupled with localization and knockout studies strongly favor the model suggesting that the Mcm2-7 hexamer is the core of the replicative helicase. However, the full replicative helicase responsible for replication in eukaryotic cells requires additional factors, including the go-ichi-ni-san (GINS) complex and Cdc45, which together form the Cdc45-MCM-GINS (CMG) complex (Figure 2). The function of the CMG complex will be discussed in detail later in this article.

## 3. Control of DNA Replication at the Replication Fork

Although the enzymatic processes of DNA replication such as unwinding, template generation/stabilization, and daughter strand synthesis are largely determined, the control of DNA replication *in vivo* is not. DNA replication requires multiple processes to coordinate and regulate highly accurate and timely duplication of genomic DNA during S phase. In addition to primase, replicative polymerases, and helicases, the DNA replication fork requires the use of accessory proteins to facilitate efficient initiation and replication fork progression. The cooperative protein complexes that participate in DNA replication are known as the replisome (Figure 2). New findings continue to suggest that the size and complexity of the replisome is greater than once thought.

### 3.1. Replication Initiation at Origins

To completely duplicate the genome in a reasonable time during the cell cycle, eukaryotic cells initiate DNA replication at multiple sites during DNA replication, whereas prokaryotic replication initiates at a single locus. Replication initiator sites are known as origins of replication (Oris) and are recognized by the origin recognition complex (ORC) of proteins in eukaryotic cells. ORCs are found associated with DNA throughout the genome and form the markers to which replication forks are recruited in a highly regulated manner (reviewed in [30] and [31]). In some eukaryotes, such as budding yeast, origins are defined by conserved nucleotide sequences, known as autonomous replication sequences (ARSs) that mark Oris. However, in most other model eukaryotes and in all metazoans, replication origins are less well defined (reviewed in [32]). Origin usage in metazoans can be dynamic, with origin firing at different sites depending on cell type and developmental stage. Nevertheless, the mechanism of replisome assembly and origin firing is highly conserved.

**Figure 3:**
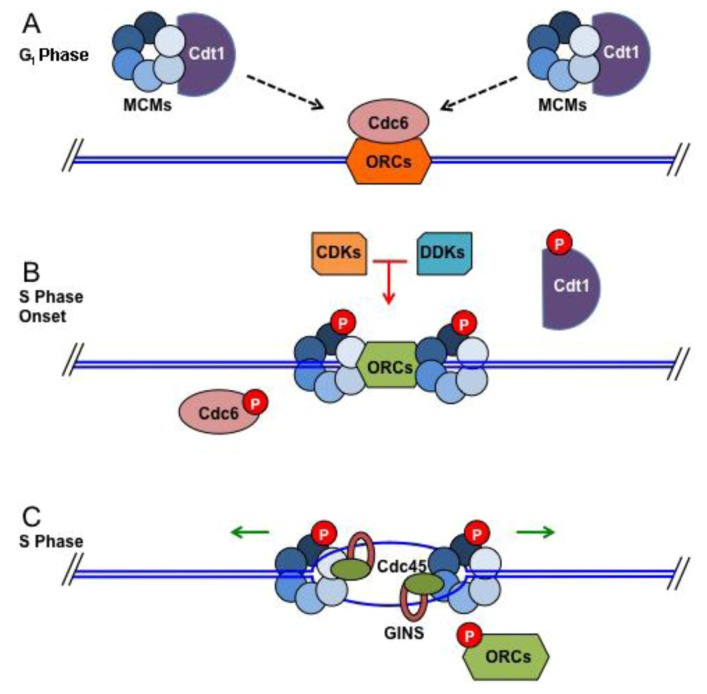
Mcm2-7 loads onto DNA at replication origins during G1 and unwinds DNA ahead of replicative polymerases. (**A**) The combined activities of Cdc6 and Cdt1 bring MCM complexes to replication origins. (**B**) CDK/DDK-dependent phosphorylation of pre-RC components leads to replisome assembly and origin firing. Cdc6 and Cdt1 are no longer required and are removed from the nucleus or degraded (**C**) MCMs and associated proteins (GINS and Cdc45 are shown) unwind DNA to expose template DNA. At this point replisome assembly can be completed and replication initiated. “P” indicates phosphorylation.

During late mitosis and G_1_ phase, Cdt1 and Cdc6 (Cdc18 in fission yeast) proteins associate with Ori sites throughout the genome and recruit Mcm2-7 (Figure 3A) [25,33,34,35,36,37]. At this time, double hexamers of the Mcm2-7 complex are loaded at replication origins [38,39]. This generates a prereplication complex (pre-RC). Origins with an associated pre-RC are considered licensed for replication. Licensed replication origins can then be “fired,” when replication actually initiates at Oris. Origin firing is brought about by multiple phosphorylation events carried out by cyclin E-CDK2 at the onset of S phase and by Cdc7-Dbf4 (DDK kinase or Dbf4-dependent kinase) prior to individual origin firing (Figure 3B) [40,41,42,43,44]. At this point, origin melting occurs and DNA unwinding by the helicase generates ssDNA, exposing a template for replication (Figure 3C). The replisome then begins to form with the localization of replisome factors such as Cdc45 [45,46]. Interestingly, the assembly of the CMG complex at origins requires the non-catalytic domain of Pol ε, suggesting a codependence between the replicative helicase and polymerase during replisome assembly [47,48]. Further recruitment assembles the remainder of replisome proteins. DNA synthesis begins on the melted template, and the replication machinery translocates away from the origin in a bidirectional manner.

To prevent multiple rounds of DNA replication during a single cell cycle, mechanisms have evolved to regulate limiting initiation factors: ORC, Cdt1, and Cdc6 (reviewed in [49]). After origin firing, Orc1, the largest ORC subunit, is ubiquitinated and degraded [50]. Phosphorylation of human Orc2 leads to the dissociation of Orc2-6 from chromatin to further prohibit re-replication, while the yeast complex appears to remain on DNA throughout the cell cycle [51,52,53]. Once phosphorylated at the onset of S phase, Cdc6 is rapidly exported from the nucleus [54]. Geminin is a specific inhibitor of Cdt1. Geminin protein levels increase throughout S phase, blocking Cdt1 from localizing to replication origins [55]. It is also well understood that Cdt1 and Cdc6 undergo rapid proteasome-dependent degradation (reviewed in [56,57]). Thus, duplicating the genome once and only once per cell cycle is controlled at the level of licensing. These multiple and redundant mechanisms prevent origin assembly to restrict replication licensing once per cell cycle. These controls emphasize the importance of preventing overreplication of chromosomal DNA, in order to preserve genomic integrity.

### 3.2. The DNA Sliding Clamp: PCNA

**Figure 4:**
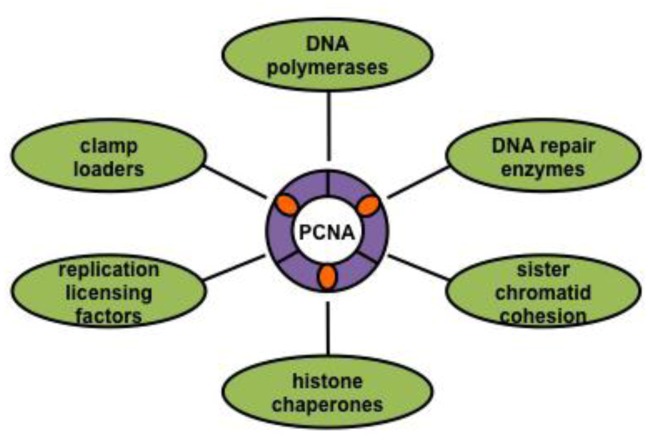
PCNA is a node of protein-protein interaction in the replisome. The PCNA homotrimer interacts with proteins in diverse processes. Many of the protein-protein interactions occur through a PCNA binding motif known as the PIP box, which binds a central domain on PCNA (orange).

DNA polymerases require additional factors to support DNA replication *in vivo.* DNA polymerases have a semiclosed hand structure, which allows them to load onto DNA and translocate. This structure permits DNA polymerase to hold the single-stranded template, incorporate dNTPs at the active site, and release the newly formed double strand. However, the conformation of DNA polymerases does not allow for their stable interaction with the template DNA. To strengthen the interaction between template and polymerase, DNA sliding clamps have evolved, promoting the processivity of replicative polymerases. In eukaryotes, this sliding clamp is a homotrimer known as proliferating cell nuclear antigen (PCNA), which form a ring structure. The PCNA ring has polarity with a surface that interacts with DNA polymerases and tethers them securely to DNA. PCNA-dependent stabilization of DNA polymerases has a significant effect on DNA replication because it enhances polymerase processivity up to 1,000-fold [58,59]. Such stimulation of replication activity makes PCNA an essential cofactor *in vivo.* PCNA also has the distinction of being one of the most common interaction platforms in the replisome to accommodate multiple processes at the replication fork [60]. Therefore, PCNA is often viewed as a regulatory cofactor for DNA polymerases (Figure 4).

Various PCNA modifications regulate the replisome through specific circumstances during DNA replication. The modifications of PCNA have dramatic effects on its function. Although there are some species-specific modifications of PCNA throughout eukaryota, the principles remain conserved. Upon DNA damage, PCNA is monoubiquitinated, which changes PCNA’s affinity from replicative polymerases to the damage-tolerant translesion synthesis (TLS) polymerases [61,62]. PCNA ubiquitination is dependent on the DNA damage checkpoint pathway and regulates dynamic changes in the replication fork. This process allows for bypass of bulky DNA damage that would otherwise prevent replication fork progression, although this method of damage bypass is error prone [62,63]. In contrast, polyubiquitination of the same site directs the cell towards DNA damage bypass by poorly characterized, but essentially error-free mechanisms [64,65,66]. PCNA can also be SUMOylated (small ubiquitin-like modifier) at the same site in yeast, and SUMOylated PCNA exists in vertebrates [67,68]. This modification is thought to suppress ubiquitination of PCNA, therefore inhibiting TLS and other DNA repair pathways, which are potentially harmful to the cell because they can introduce mutations and genome rearrangements [69,70,71]. In addition, acetylation of PCNA may play a role in enhancing the processivity of associated polymerases [72]. These findings indicate that PCNA modifications play critical roles in controlling pathway selection for DNA damage management during DNA replication.

### 3.3. Clamp Loaders

PCNA fully encircles DNA and must be loaded onto DNA at the replication fork. At the leading strand, this is a relatively infrequent process, because DNA replication is essentially continuous until replication is terminated. However, at the lagging strand, Pol δ is continually loaded at the start of each Okazaki fragment. This constant initiation of Okazaki fragment synthesis requires repeated PCNA loading for efficient DNA replication. PCNA loading is accomplished by the replication factor C (RFC) complex, which is comprised of five AAA^+^ ATPases [73]. RFC recognizes primer-template junctions and loads PCNA at these sites [74,75]. The PCNA homotrimer is opened by RFC using energy from ATP hydrolysis and is then loaded onto DNA in the proper orientation to facilitate its association with the polymerase [76,77]. Clamp loaders can also unload PCNA from DNA, a mechanism that becomes important when replication must be terminated [77].

Eukaryotes have evolved multiple clamp loading complexes, each of which appears to function in a separate pathway. The canonical clamp loader essential for DNA replication is RFC and includes Rfc1, Rfc2, Rfc3, Rfc4 and Rfc5. At least three RFC-like complexes exist in eukaryotic cells. RFC^Ctf18^, which contains Ctf18 in place of Rfc1, promotes sister chromatid cohesion and regulates replication speed [78,79,80,81]. RFC^Elg1^, which contains Elg1, is thought to unload SUMOylated PCNA in the presence of DNA damage to allow for replication progression through damaged DNA templates [71]. The RFC^Rad17/Rad24^ clamp does not load PCNA, but loads the 9-1-1 complex at DNA damage sites during the replication checkpoint response [82]. Thus, DNA replication can be regulated at the level of PCNA clamp loading, in order to accommodate multiple processes that take place during DNA replication (Figure 4).

### 3.4. Okazaki Fragment Maturation

DNA replication on the lagging strand is discontinuous. At the end of Okazaki fragment synthesis, Pol δ runs into the previous Okazaki fragment and displaces its 5' end containing the RNA primer and a small segment of DNA. This generates an RNA-DNA single strand flap, which must be cleaved, and the nick between the two Okazaki fragments must be sealed by DNA ligase I. This process is known as Okazaki fragment maturation and can be handled in two ways: one mechanism is designed to process short flaps, while the other deals with long flaps [9]. Pol δ is able to displace up to 2 to 3 nucleotides of DNA or RNA ahead of its polymerization, generating a short “flap” substrate for Fen1, which can remove nucleotides from the flap, about one nucleotide at a time. By repeating cycles of this process, Pol δ and Fen1 can coordinate the removal of RNA primers and leave a DNA nick at the lagging strand [83]. It has been proposed that this iterative process is preferable to the cell because it is tightly regulated and does not generate large flaps that need to be excised [84]. In the event of deregulated Fen1/Pol δ activity, the cell uses an alternative mechanism to generate and process long flaps by using Dna2, which has both helicase and nuclease activities [85]. Dna2 is localized to Okazaki fragments [86,87], and importantly, Dna2 is thought to act on Okazaki fragment ends that are not optimal for Fen1 activity [88]. For example, DNA flaps containing secondary structure and long flaps are both poor Fen1 substrates. The nuclease activity of Dna2 is required for removing these long flaps, leaving a shorter flap to be processed by Fen1. Electron microscopy studies indicate that nucleosome loading on the lagging strand occurs very close to the site of synthesis [89]. Thus, Okazaki fragment maturation is an efficient process that occurs immediately after the nascent DNA is synthesized. These redundant and proficient mechanisms showcase the importance of Okazaki fragment maturation in protecting genomic integrity.

### 3.5. Linking Helicase and Polymerase Functions

The DNA helicases and polymerases must remain in close contact at the replication fork (Figure 2). If unwinding occurs too far in advance of synthesis, large tracts of ssDNA are exposed. This can activate DNA damage signaling or induce aberrant DNA repair processes. To thwart these problems, the eukaryotic replisome contains specialized proteins that are designed to regulate the helicase activity ahead of the replication fork. These proteins also provide docking sites for physical interaction between helicases and polymerases, thereby ensuring that duplex unwinding is coupled with DNA synthesis.

There is evidence that Cdc45 has such a role. It has been shown that Cdc45 coordinates MCM progression with the replisome [90]. Before DNA replication initiates, S-phase kinase DDK phosphorylates Mcm4, which allows stable Cdc45-MCM complex formation. This is an important step in replisome assembly at origins and is essential for origin firing [91]. Furthermore, during S phase, Cdc45 depletion prevents DNA replication stress signaling through downregulation of active DNA synthesis, reinforcing the notion that Cdc45 is an important regulatory protein, required for DNA replication [92].

During the past decade, a protein complex termed GINS has drawn much attention. The GINS complex is comprised of the Sld5 (go), Psf1 (ichi), Psf2 (ni), and Psf3 (san) proteins and (named for 5, 1, 2, 3 in Japanese) was originally identified in budding yeast and in *Xenopus laevis* extracts [93,94]. GINS has been shown to regulate the interaction of the MCM subunits with Cdc45 [95]. The high-molecular-weight complex consisting of the MCM, GINS, and Cdc45 is now known as the CMG complex and can be observed in human cells [96,97]. The GINS complex may provide additional regulation of the MCM helicase during replication progression and in response to DNA replication stress. Importantly, the full CMG complex is required for DNA unwinding, demonstrating that the complex of Cdc45-MCM-GINS is the functional DNA helicase machinery in eukaryotic cells (Figure 2) [98,99]. It is also worth noting that the Psf3 subunit is phosphorylated by DNA damage signaling kinases ATM and ATR, suggesting a possible mechanism of MCM regulation upon DNA damage during DNA replication [100].

While the identification of the CMG complex adds to our understanding of the regulation of the MCM helicase activity, how the CMG complex interacts with the bulk of the replisome is still emerging. Recent studies have shown that the Ctf4/And1 protein interacts with both the CMG complex and Pol α [101,102]. Ctf4 is a Pol α accessory factor, which is required for the recruitment of Pol α to replication origins [103]. Indeed, human Ctf4/And1 stimulates Pol α function *in vitro*, whereas depletion of the protein greatly reduces replication fork progression rates in human cells [101]. Therefore, the interaction of CMG and Ctf4 suggests the critical role of CMG in tethering Pol α to the replication fork. Considering that Pol α is continually required on the lagging strand and that CMG has helicase activity, CMG may couple DNA unwinding with primer synthesis on the lagging strand. Such a coupling mechanism may help to minimize the length of the exposed ssDNA tract at the replication fork, thereby promoting efficient DNA replication and ensuring genomic integrity.

How, then, is leading-strand synthesis coupled with unwinding activity? Mrc1/Claspin appears to be the best candidate for this task. In budding yeast, Mrc1 interacts with Pol ε as well as MCM proteins in unperturbed S phase and under genotoxic stress [104]. The importance of this direct link between the helicase and the leading-strand polymerase is underscored by results in cultured human cells, where Mrc1/Claspin is required for efficient replication fork progression [105]. These results suggest that efficient DNA replication also requires the coupling of helicases and leading-strand synthesis.

Taken together, it appears that the CMG complex has a central role in coupling template unwinding with DNA polymerases. Importantly, CMG interacts with both leading- and lagging-strand polymerases via Mrc1/Claspin and Ctf4/And-1, respectively (Figure 2). This setup also helps coordinate leading- and lagging-strand synthesis, integrating required processes at the replication fork into a centralized replisome. In this model, protein-protein interactions allow for crosstalk among multiple enzymatic processes essential for DNA replication. For instance, leading-strand synthesis can be restrained to closely match the rate of the lagging-strand synthesis. Considering that replication efficiency is reduced when Ctf4/And-1 or Mrc1/Claspin is lost, the coordination of multiple activities plays a critical role in replisome progression.

Additional factors are required for maintaining proper replisome structure and function. One such factor is the fork protection complex (FPC), which is comprised of Timeless and Tipin (Tof1-Csm3 in budding yeast and Swi1-Swi3 in fission yeast). FPC is a replisome component and appears to play a key role in efficient DNA replication (reviewed in [106]). As neither FPC protein appears to have any enzymatic function, it is likely that their role is to maintain or stabilize the replisome during DNA replication. Indeed, in the absence of functional FPC proteins, DNA replication takes longer to complete and results in shorter replication tracts, indicating that the FPC is required for efficient replisome progression [107,108]. In addition, the FPC has been shown to associate with nonreplisome proteins involved in multiple genome maintenance mechanisms, suggesting that the FPC mediates interactions between the replisome and other activities that take place on DNA during replication (reviewed in [106]). Given that loss of Claspin, Ctf4/And-1, and the FPC leads to inefficient replication, maintaining proper protein-protein interactions in the replisome is vital to coordinating the variety of processes occurring at the replication fork during DNA replication.

### 3.6. Replication Checkpoint Proteins

In order to preserve genetic information every time the cell divides, DNA replication must be completed with high fidelity. To achieve this task, eukaryotic cells are equipped with genome surveillance systems that detect errors or problems during DNA replication. Proteins required for this mechanism, termed the DNA replication checkpoint, have several mechanisms to preserve genomic integrity. To ensure that DNA is faithfully repaired and replicated, checkpoint proteins function to arrest the cell cycle to allow time for DNA repair and prevent cells from entering mitosis. Checkpoint proteins also directly facilitate some DNA repair pathways, while they stabilize replication fork structures to prevent further damage. These mechanisms are essential to avoid passing down mutations or other chromosome aberrations to offspring.

The checkpoint proteins are well conserved throughout the domain Eukaryota. At the central node of the checkpoint networks are two phosphatidyl inositotide-3' kinase (PI3K)-like kinases, ATR and ATM. ATM and ATR share a consensus target phosphorylation sequence, the SQ/TQ motif. However, their targets and their roles in the cells differ substantially. ATR is an essential mammalian gene, whereas ATM is not [109,110]. This is thought to be due to the specificity of each kinase for different types of DNA damage. ATM arrests the cell cycle in response to DNA double strand breaks (DSBs), which are a relatively rare event in the cell. Conversely, ATR and its obligate checkpoint partner, ATR-interacting-protein (ATRIP), are responsive to stretches of ssDNA that are coated by RPA (Figure 2) [111,112]. Importantly, ssDNA accumulation occurs frequently during DNA replication. Long stretches of ssDNA are also present during replication stress. Therefore, ATR-ATRIP is heavily relied upon to arrest the cell cycle and to preserve genome integrity during DNA replication. Indeed, ATR is found on chromatin during S phase in a manner similar to RPA and the checkpoint mediator Claspin [113]. Moreover, studies in budding yeast demonstrated that Mec1 (the ATR homologue) is required for normal DNA synthesis during S phase and that *mec1* deficiency causes dramatically longer S phase [114]. These findings reinforce the importance of checkpoint proteins during S phase.

The generation of ssDNA tracts is important in initiating the checkpoint pathways downstream of replication-associated damage. Once sufficiently long, RPA coated ssDNA stretches are able to recruit ATR-ATRIP [112,115]. However, to become fully active, the ATR kinase relies on sensor proteins that “sense” whether the checkpoint proteins are localized to a valid site of DNA replication stress. The 9-1-1 (Rad9-Hus1-Rad1) heterotrimeric clamp and its clamp loader RFC^Rad17^ recognize the damaged DNA. It has been demonstrated *in vitro* that RFC^Rad17^ recognizes gapped or nicked DNA and loads the 9-1-1 clamp onto DNA [82]. The presence of 9-1-1 on DNA is sufficient to facilitate the interaction between ATR-ATRIP and a group of proteins termed checkpoint mediators, such as TopBP1 and Mrc1/Claspin. TopBP1 interacts with (and recruits) the phosphorylated Rad9 component of 9-1-1 and binds ATR-ATRIP, which phosphorylates Chk1 [116,117,118,119,120]. Furthermore, Mrc1/Claspin is required for the complete activation of ATR-ATRIP that phosphorylates Chk1, the major downstream checkpoint effector kinase [121,122,123]. Claspin is a component of the replisome and contains a domain for docking with Chk1, revealing a specific function of Claspin during DNA replication: the promotion of checkpoint signaling at the replisome (Figure 2) [124].

Chk1 signaling is vital for arresting the cell cycle and preventing cells from entering mitosis with incomplete DNA replication or DNA damage. In metazoans, active Chk1 induces a robust G_2_/M checkpoint by phosphorylating Cdc25A protein, a phosphatase that removes inhibitory phosphorylations on Cdk1 and Cdk2. Once phosphorylated, Cdc25A is targeted for proteasome-dependent degradation by the ubiquitin ligase complex containing the F-box protein β-TrCP [125]. Thus, Chk1 maintains inhibitory phosphorylation of metazoan Cdk1 and Cdk2 to block mitotic progression and origin firing, respectively [126,127,128]. The Chk1-dependent Cdk inhibition may constitute the single most important function of the ATR-Chk1 checkpoint, to arrest the cell cycle and allow sufficient time for completion of DNA repair mechanisms, which in turn prevents the inheritance of damaged DNA. In addition, Chk1-dependent Cdk inhibition plays a critical role in inhibiting origin firing during S phase. This mechanism prevents continued DNA synthesis and is required for the protection of the genome in the presence of replication stress and potential genotoxic conditions [129,130,131]. Thus, ATR-Chk1 activity further prevents potential replication problems at the level of single replication origins by inhibiting initiation of replication throughout the genome, until the signaling cascade maintaining cell-cycle arrest is turned off.

### 3.7. Replication through Nucleosomes

**Figure 5:**
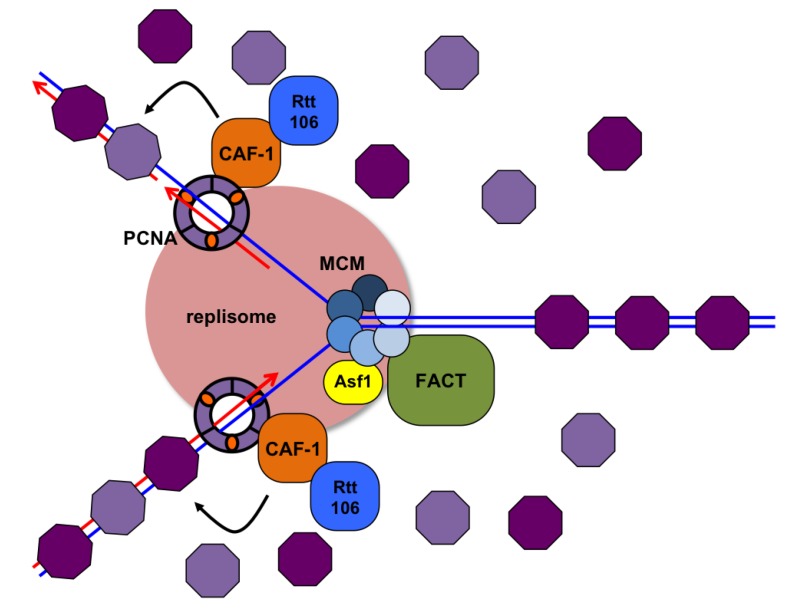
Nucleosome displacement and deposition during DNA replication. Histones are removed from chromatin ahead of the replication fork. FACT may facilitate this process. Asf1 recruits histone H3-H4 dimers to the replication fork. CAF-1 and Rtt106 load newly synthesized (light purple) histones to establish chromatin behind the fork. Previously loaded histones (dark purple) are also deposited on both daughter DNA strands. The histone chaperones involved in these processes are associated with replisome proteins: CAF-1/Rtt106 with PCNA and FACT/Asf1 with MCMs.

Eukaryotic genomes are substantially more complicated than the smaller and unadorned prokaryotic genomes. Eukaryotic cells have multiple noncontiguous DNA components, chromosomes, each of which must be compacted to allow packaging within the confined space of a nucleus. Chromosomes are packaged by wrapping ~147 nucleotides (at intervals averaging 200 nucleotides) around an octamer of histone proteins, forming the nucleosome [132,133,134,135]. The histone octamer includes two copies each of histone H2A, H2B, H3, and H4. Histone proteins are subject to a variety of modifications, including phosphorylation, acetylation, methylation, and ubiquitination that represent vital epigenetic marks. The tight association of histone proteins with DNA in nucleosomes suggests that eukaryotic cells possess proteins that are designed to remodel histones ahead of the replication fork, in order to allow smooth progression of the replisome. It is also essential to reassemble histones behind the fork to reestablish the nucleosome confirmation. Furthermore, it is important to transmit the epigenetic information found on the parental nucleosomes to the daughter nucleosomes, in order to preserve the same chromatin state. In other words, the same histone modifications should be present on the daughter nucleosomes as were on the parental nucleosomes. This must all be done while doubling the amount of chromatin, which requires incorporation of newly synthesized histone proteins. This process is accomplished by “histone chaperones” and “histone remodelers,” which are discussed below (Figure 5).

Several histone chaperones are known to be involved in replication-coupled nucleosome assembly. The FACT complex components were originally identified as proteins that greatly stimulate transcription by RNA polymerase II (reviewed in [136]). In budding yeast, FACT was found to interact with DNA Pol α-primase complex, and the FACT subunits were found to interact genetically with replication factors [137,138,139]. More recently, studies showed that FACT facilitates DNA replication *in vivo* and is associated with the replisome in budding yeast and human cells [95,140,141,142]. The FACT complex is a heterodimer that does not hydrolyze ATP, but facilitates the “loosening” of histones in nucleosomes [143]. How the FACT complex relieves the tight association of histones for DNA in nucleosomes is unanswered. Further, the mechanisms and factors facilitating nucleosome removal during DNA replication remain unknown.

Another histone chaperone associating with the replisome is Asf1, which interacts with MCM in a manner dependent on histone H3-H4 dimers [144]. It is hypothesized that Asf1 can pass newly synthesized H3-H4 dimers to deposition factors behind the replication fork [145,146,147]. This activity makes histone H3-H4 dimers available at the site of histone deposition just after replication (Figure 5). Another highly related problem for the replication fork in the context of chromatin is the deposition of histones on nascent DNA. Chromatin formation proteins deposit histones onto both newly replicated DNA strands to form chromatin [148]. The heterotrimeric chaperone chromatin assembly factor 1 (CAF-1) is involved in this mechanism [149,150]. CAF-1 contains a PCNA-binding motif called PIP-box, allowing CAF-1 to associate with the replisome through PCNA and to deposit histone H3-H4 dimers onto newly synthesized DNA [151,152] (Figure 5). The Rtt106 chaperone also participates in this process, associating with CAF-1 and H3/H4 dimers during chromatin formation [153,154]. These processes load newly synthesized histones onto DNA. How the existing chromatin marks are transferred from parental to daughter strands is not characterized. Daughter strands contain H3-H4 from parental DNA, but the mechanism of histone transfer is a continuing field of study [155].

At this point, nucleosomes form by the association of histone H2A/H2B with the deposited histone H3-H4. This process may occur through the FACT complex, since it is associated with the replisome and binds free H2A-H2B; or possibly another histone H2A-H2B chaperone, Nap1 [156]. Electron microscopy studies show that this occurs very quickly, as nucleosomes can be observed forming just a few hundred base pairs after the replication fork [157]. Therefore, the entire process of forming new nucleosomes takes place just after replication due to the coupling of histone chaperones to the replisome.

## 4. Challenges to Replication

**Figure 6:**
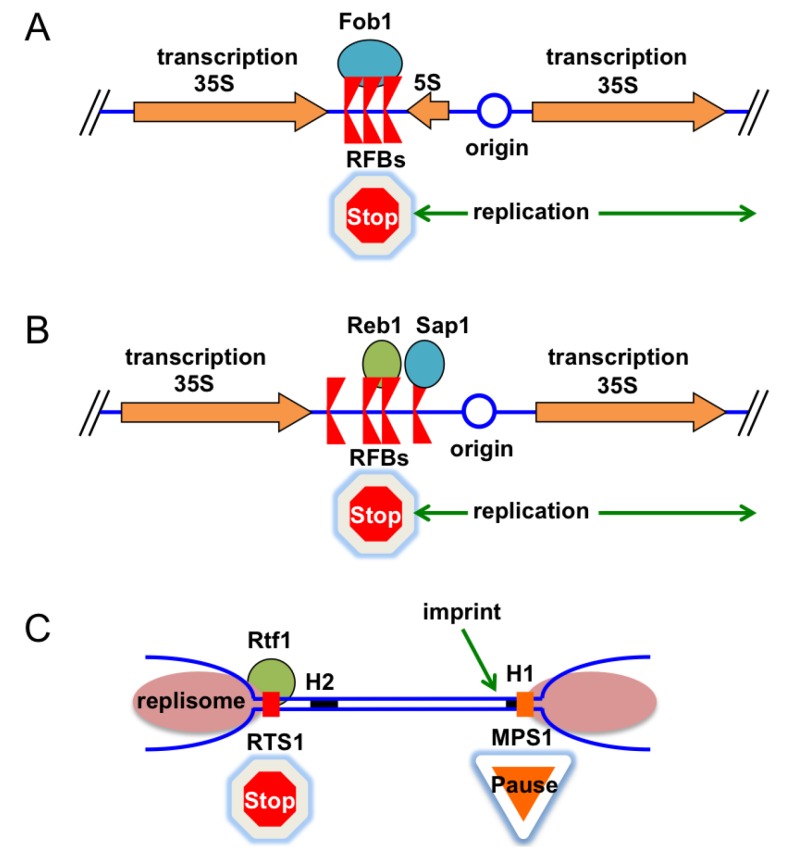
Replication Fork Barriers (RFBs) control DNA progression to protect genomic integrity. RFBs allow for the coordination of DNA replication with important processes on chromatin. (**A**) In budding yeast, Fob1 blocks replication fork progression in the opposing direction to 35S ribosome transcription. (**B**) In fission yeast, collisions between the transcription machinery and the replication fork are prevented by Reb1 and Sap1, which bind to the RFBs and block replisome progression near the 3' end of the 35S ribosome genes. (**C**) At the mating-type (*mat1*) locus in fission yeast, Rtf1 bound to the *RTS1* site prevents replication fork progression at the 5' side of *mat1*. Only the fork in the 3' direction can progress through the region. This fork pauses at *MPS1* and generates an imprint, probably a DNA strand discontinuity, which is required for a specific recombination event that allows for mating-type switching.

The varied chromatin landscape within the eukaryotic genome requires a “toolkit” of proteins that facilitate replisome progression through a variety of genomic regions that are difficult to replicate. Defects in this mechanism result in replication fork collapse and lead to genomic instability. In this section, analysis will focus on a subset of challenging genomic regions for DNA replication (Figure 6). It has become apparent that specialized mechanisms have evolved for the replisome to replicate DNA through these regions.

### 4.1. Replication Fork Barriers

In prokaryotes, such as the *Escherichia coli* bacterium, bidirectional replication initiates at a single replication origin on the circular chromosome and terminates at a site approximately opposed from the origin [158]. This replication terminator region contains DNA sequences known as *Ter* sites, polar replication terminators that are bound by the Tus protein. The *Ter*-Tus complex counteracts helicase activity, resulting in replication termination [159]. In this way, prokaryotic replication forks pause and terminate in a predictable manner during each round of DNA replication.

In eukaryotes, the situation differs. Replication termination typically occurs by the collision of two replication forks anywhere between two active replication origins. The location of the collision can vary based on the replication rate of each of the forks and the timing of origin firing. Often, if a replication fork is stalled or collapsed at a specific site, replication of the site can be rescued when a replisome traveling in the opposite direction completes copying the region. However, there are numerous programmed replication fork barriers (RFBs) and replication “challenges” throughout the genome. To efficiently terminate or pause replication forks, some fork barriers are bound by RFB proteins in a manner analogous to *E. coli* Tus [158]. In these circumstances, the replisome and the RFB proteins must specifically interact to stop replication fork progression.

### 4.2. Fork Barriers at rDNA Arrays in Eukaryotic Cells

Certain RFB proteins function to prevent collisions between replication forks and the RNA transcription machinery. One of the best characterized RFBs is situated near the 3' end of the 35S ribosomal RNA gene (rDNA) in budding yeast [160]. Budding yeast chromosome XII contains an array of approximately 150 rDNA repeats, which are highly transcribed in order to produce the ribosomes necessary for translation. Therefore, transcription forks are exceedingly common in rDNA regions, which can lead to head-on collisions between replication and transcription forks, possibly leading to collapse of one or both forks. To prevent these collisions, replisome progression opposing the direction of ribosome transcription is inhibited at rDNA repeats, thus allowing for replication and transcription to proceed in the same direction through the rDNA loci [161]. How is this polar replication block achieved?

In budding yeast, the Fob1 protein is responsible for the rDNA barrier (Figure 6A). Fob1 binds RFBs located at the 3' non-transcribed region of each rDNA gene [162]. Fob1 operates by looping or wrapping the DNA around itself to dramatically alter the local chromatin state and block replication from the 3' direction [163]. This Fob1-dependent chromatin structural change not only causes polar replication fork pausing but also leads to increased recombination at the RFB region [164]. Additional factors such as Tof1 and Csm3, components of the FPC are required to ensure stable fork pausing at rDNA loci. Tof1 and Csm3 appear to work together with Fob1 to counteract Rrm3, a DNA helicase required for replication through the rDNA RFB regions. It is proposed that Rrm3 displaces Fob1 to facilitate fork progression [165]. In addition, Rrm3 is thought to be required for replication restart in other genomic loci requiring displacement of nonhistone proteins from DNA [166,167,168,169,170]. Interestingly, Rrm3 translocates with the replication fork and physically interacts with PCNA [171,172]. Therefore, similar protein-protein interactions between RFB proteins and Rrm3 may allow for polar passage of replication forks through multiple fork block sites throughout the genome.

Although Fob1 is a budding yeast-specific protein, similar 3' specific replication termination occurs in the rDNA array in fission yeast and mammalian cells. The fission yeast rDNA unit has four RFBs (*Ter1-4*), three of which are known to use RFB proteins and the FPC to achieve polar fork arrest (Figure 6B). The DNA binding protein Sap1, which also recognizes the mating-type locus, causes polar fork arrest at *Ter1*, while fork arrest at *Ter2* and *Ter3* is regulated by another RFB protein, Reb1, which is homologous to mammalian transcription terminator factor 1 (TTF-1) [173,174,175,176]. Human TTF-1 also localizes to the rDNA RFB. TTF-1 performs “double duty” at the RFB: TTF-1 promotes polar replication fork arrest and also mediates transcription termination at the rDNA, preventing rDNA transcription machinery from running past the RFB [177,178]. Interestingly, fission yeast Reb1 and mammalian TTF-1 share the Myb-like DNA-binding domain, which is critical for DNA binding and protein function [179]. These proteins act to specifically inhibit polar replication progression, coordinating DNA replication with transcription to minimize fork collisions and genome instability.

### 4.3. Replication Termination at the Fission Yeast Mating-Type Locus

In addition to site-specific fork pausing required to prevent collision between replication and transcription machinery, fork pausing also allows for programmed cellular events that are coordinated with replication of specific genomic loci. For example, the fission yeast genome contains two genetically programmed fork pausing sites near the mating-type (*mat1*) locus: the *mat1* pausing sites 1 (*MPS1*) and replication termination site 1 (*RTS1*). A strong polar fork arrest at *RTS1* blocks one replication fork moving into the *mat1* locus, allowing only the opposing fork to migrate into the *mat1* locus. This fork pauses at *MPS1*, generating an imprint that initiates a replication-coupled recombination event, leading to mating-type switching (Figure 6C) [180,181,182,183,184,185].

The *RTS1*-dependent fork arrest is DNA sequence-specific, because inserting the *RTS1* sequence into other genomic loci in *Schizosaccharomyces pombe* also results in polar fork arrest and increased recombination at those sites [186,187]. The *RTS1* DNA sequence was used to identify a site-specific replication terminator factor, Rtf1. Rtf1 contains an Myb-like DNA-binding domain and directly binds DNA to block replisome progression. Rtf1 mutations disrupt polar fork arrest, indicating that Rtf1 is required for unidirectional replication termination at the *RTS1* loci [188,189]. Interestingly, efficient pausing at *RTS1* also requires the presence of specific replisome proteins. The Pol α gene (*swi7*) in *S. pombe* is required to initiate recombination at the *mat1* locus. Additionally, the *swi1* and *swi3* genes, encoding the fission yeast FPC subunits, are required for both *MPS1*-pausing and *RTS1*-fork arrest [190,191]. The requirement for both replication termination factors on the DNA and replisome components suggests that these proteins may interact to regulate replication pausing or termination at the *mat1* locus. Together, these RFB sites provide insights into the role of chromatin-bound proteins in regulating replisome progression at specific loci.

### 4.4. Replication Slow Zones

Examining DNA replication on a global level, it appears that some loci are more sensitive to DNA replication stress than others. Analysis of chromosomal DNA using two-dimensional agarose gel electrophoresis, showed that checkpoint mutants accumulate replication forks at specific genomic regions [114]. This led to the suggestion that certain genomic regions take longer to replicate because they are more difficult to replicate when compared with the rest of the genome. The replication fork-enriched loci were coined replication slow zones (RSZs). DNA replication through RSZs requires the checkpoint protein Mec1 (the budding yeast ATR homologue), implying that checkpoint pathways play an important role during replication of RSZs [114]. This is consistent with the observation that functional checkpoint proteins are generally required to replicate DNA during stress [192]. Early hypotheses proposed that RSZs were advantageous for general replication, allowing for the replisome to coordinate with other processes (e.g., slowing replication to enhance chromatid pairing or nucleosome loading) [114]. Recent work has suggested that *mec1* mutant cells fail to replicate through RSZs due to a partial inability to upregulate dNTP levels (relieving inhibition of ribonucleotide reductase II by Sml1). This paucity of dNTPs leads to replication fork collapse in RSZs, because replication of RSZs requires higher local levels of dNTPs [193,194]. Therefore, the primary function of Mec1 may be to act as a sensor at the replication fork, upregulating nucleotide synthesis when there is a demand for increased dNTP levels during DNA replication. Considering that the checkpoint is required even in the absence of exogenous replication stressors, something inherent to RSZs causes replication stress.

As described above, DNA replication through these challenging regions requires checkpoint proteins in budding yeast. This is consistent with replication in the presence of exogenous genotoxic agents such as methyl methanesulphonate (MMS: an alkylating agent, which arrests forks) or hydroxyurea (HU: a ribonucleotide reductase II inhibitor, which depletes cellular dNTP pools and arrests forks). In the absence of Rad53 checkpoint kinase, budding yeast cells accumulate unusual replication fork structures in response to MMS or HU; thus cells are unable to complete replication [195,196]. In contrast, wild-type cells are eventually able to complete replication in the presence of genotoxic agents, albeit slowly and with continued activation of repair and checkpoint pathways.

**Figure 7:**
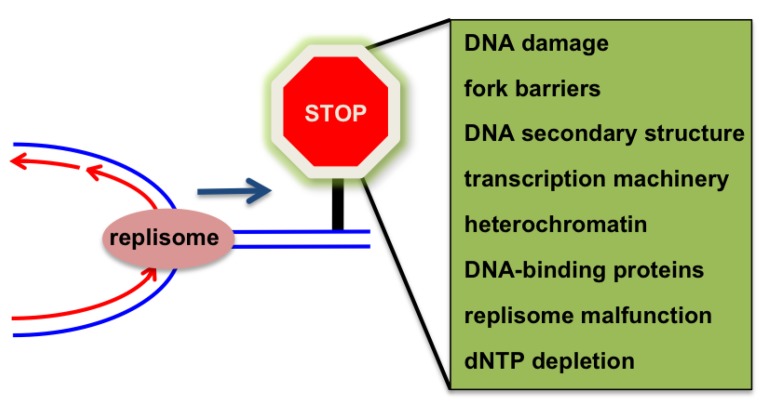
Replication stress is induced by multiple factors *in vivo.* Some stresses are relatively rare events such as DNA damage, while others must be encountered at the same genomic sites every S phase, such as difficult-to-replicate regions.

In MMS-treated cells, DNA replication stress is induced globally, and replication is generally slowed by the checkpoints. In the RSZ model, the stress is induced by replication of the specific genomic region. The replisome encounters difficulties at these regions, resulting in fork arrest and generation of DNA structures that are recognized by checkpoint proteins. Whether the source of replication stress is exogenous or endogenous, checkpoint proteins recognize stalled forks, upregulate nucleotide synthesis, activate repair pathways, and arrest the cell cycle. By associating with the replisome, checkpoint kinases are well positioned to send signals to cope with fork arrest in response to a multitude of replication stressors, including endogenous obstacles on DNA sequences and exogenous genotoxic agents (Figure 7).

### 4.5. Fragile Sites

Recurrent DNA breaks that can be visualized on metaphase chromosomes as breaks and gaps are known as fragile sites. Fragile sites lead to recurrent breaks in families and were initially characterized as DNA breakage sites inherited in a manner predicted by Mendelian genetics; these breaks could be visualized on metaphase chromosomes and mapped to specific chromosome arm regions [197]. Further investigations have connected the occurrence of fragile sites with human diseases such as fragile X syndrome, the most common heritable form of mental retardation [198]. In cultured cells from normal individuals, these sites are relatively stable, and chromosome breakage rarely occurs. Therefore, these sites are known as “rare” fragile sites in normal cells. However, breakage at these sites can be induced by depleting folate or by adding excess thymidine in cultured human cell lines [199,200,201]. Because excess thymidine induces DNA replication stress, these findings suggest that DNA breaks at rare fragile sites are induced by DNA replication problems. Such expression of “fragile sites” (*i.e.*, breakage at rare fragile sites) often occurs in chromosome regions associated with nucleotide repeat expansion. Because nucleotide repeats are considered to be poor templates for DNA polymerases and can induce replication fork stalling, breakage at rare fragile sites are likely to occur during DNA replication [202,203,204].

By treating normal cells with various replication inhibitors, investigators identified additional breakage sites, known as “common” fragile sites (reviewed in [205]). Low doses of aphidicolin (a Pol α inhibitor) lead to consistently observed DNA breaks at over 70 genomic sites [206]. Other studies have reported additional fragile sites using bulky nucleotide analogues, such as 5-aza-cytidine or bromodeoxyuridine [207]. These sites are less well characterized than aphidicolin-dependent fragile sites, but both types of fragile sites are found in AT-rich genomic loci. Thus, chromosome breakage at specific loci is often due to replication perturbations, which are generally provoked by inherent features associated with the specific chromosomal loci.

Fragile sites appear to share common features that may help to understand the mechanisms of chromosome breakage. Breakage rates at aphidicolin fragile sites are dependent on drug concentrations, and these sites tend to form secondary structures and replicate late in S phase [208,209,210]. Because common fragile sites occur in AT-rich repeat regions, it is possible that the properties of AT-rich DNA hinder proper replication processes. For example, AT-repeat rich microsatellites contain sequences that are structurally flexible with regard to base pairing and can form hairpin secondary structures [211]. Perhaps during DNA replication, while DNA is unwound and ssDNA is exposed at fragile sites, the flexible DNA sequences allow for the formation of hairpin structures, thus preventing replication fork progression and in some cases leading to DNA breaks. Interestingly, all fragile sites (including common and rare) share a late-replicating phenotype [205]. It is possible that late S-phase replication of fragile sites allows for proper DNA replication of difficult templates. However, conversely, it is also possible that cells delay replication of fragile sites owing to the inherent difficulty of replicating the region. As a consequence, these fragile sites may break due to diminished dNTP pools at the end of S phase, thus requiring checkpoint proteins to complete the region.

Other factors may play a role in the breakage of fragile sites. When a fragile region sequence was deleted from human/mouse hybrid cells, the rate of DNA breakage at the site was reduced 2- to 12-fold [212]. However, DNA breakage at the site was not entirely abrogated, implying that chromatin context, not simply DNA sequence, also contributes to breakage propensity. Additionally, ATR has been shown to be required for replication through these regions, suggesting intimate regulation of DNA replication through these regions by the cell cycle checkpoint [213]. These studies indicate that a combination of DNA sequence, chromatin state, and the replication timing of a specific locus contributes to the “difficulty” of replication in the fragile sites. Global mapping and characterizations of these sites will reveal how various features of fragile sites contribute to their challenging replication phenotype.

## 5. Conclusions and Closing Remarks

The study of eukaryotic DNA replication has continued to expand over the past several decades and will likely continue to do so, filling the knowledge gaps in the regulation of replication processes. Many of the recent advances in the field have focused on how specific DNA sequences, structures, and regions are specifically regulated. Further studies will elucidate how characterized and yet-unidentified replisome proteins contribute to replication processes in a site-specific manner. In our current understanding, the replisome and replisome-associated factors are able to respond to a variety of hindrances to successfully complete DNA replication (Figure 7). The above review of challenging loci for replication is by no means exhaustive, and new techniques will likely identify even more genome regions that require specialized mechanisms for successful and efficient replication. Genome-wide approaches will allow us to understand the genomic regions and features affected by perturbation of specific replisome components. Current developments in single-molecule studies will open opportunities to test mechanistic models of replication in a loci-specific manner. The next few years should see great advances in our understanding of replication regulation at the global and locus level. Future studies will uncover the mechanisms by which checkpoint proteins and replisome-interacting factors cooperate together to ensure replisome progression of difficult-to-replicate genomic regions.

## References

[1] Meselson M., Stahl F.W. (1958). The replication of DNA in *Escherichia coli*. Proc. Natl. Acad. Sci. USA.

[2] Bessman M.J., Lehman I.R., Simms E.S., Kornberg A. (1958). Enzymatic synthesis of deoxyribonucleic acid. II. General properties of the reaction. J. Biol. Chem..

[3] Lehman I.R., Bessman M.J., Simms E.S., Kornberg A. (1958). Enzymatic synthesis of deoxyribonucleic acid. I. Preparation of substrates and partial purification of an enzyme from *Escherichia coli*. J. Biol. Chem..

[4] Okazaki R., Okazaki T., Sakabe K., Sugimoto K., Sugino A. (1968). Mechanism of DNA chain growth. I. Possible discontinuity and unusual secondary structure of newly synthesized chains. Proc. Natl. Acad. Sci. USA.

[5] Wold M.S., Kelly T. (1988). Purification and characterization of replication protein A, a cellular protein required for *in vitro* replication of simian virus 40 DNA. Proc. Natl. Acad. Sci. USA.

[6] Alani E., Thresher R., Griffith J.D., Kolodner R.D. (1992). Characterization of DNA-binding and strand-exchange stimulation properties of y-RPA, a yeast single-strand-DNA-binding protein. J. Mol. Biol..

[7] Siegal G., Turchi J.J., Myers T.W., Bambara R.A. (1992). A 5' to 3' exonuclease functionally interacts with calf DNA polymerase ε. Proc. Natl. Acad. Sci. USA.

[8] Goulian M., Richards S.H., Heard C.J., Bigsby B.M. (1990). Discontinuous DNA synthesis by purified mammalian proteins. J. Biol. Chem..

[9] Waga S., Bauer G., Stillman B. (1994). Reconstitution of complete SV40 DNA replication with purified replication factors. J. Biol. Chem..

[10] Budd M., Campbell J.L. (1987). Temperature-sensitive mutations in the yeast DNA polymerase I gene. Proc. Natl. Acad. Sci. USA.

[11] Sitney K.C., Budd M.E., Campbell J.L. (1989). DNA polymerase III, a second essential DNA polymerase, is encoded by the *S. cerevisiae CDC2* gene. Cell.

[12] Boulet A., Simon M., Faye G., Bauer G.A., Burgers P.M. (1989). Structure and function of the *Saccharomyces cerevisiae CDC2* gene encoding the large subunit of DNA polymerase III. EMBO J..

[13] Morrison A., Araki H., Clark A.B., Hamatake R.K., Sugino A. (1990). A third essential DNA polymerase in *S. cerevisiae*. Cell.

[14] Fisher P.A., Wang T.S., Korn D. (1979). Enzymological characterization of DNA polymerase α. Basic catalytic properties processivity, and gap utilization of the homogeneous enzyme from human KB cells. J. Biol. Chem..

[15] Nethanel T., Zlotkin T., Kaufmann G. (1992). Assembly of simian virus 40 Okazaki pieces from DNA primers is reversibly arrested by ATP depletion. J. Virol..

[16] Waga S., Stillman B. (1994). Anatomy of a DNA replication fork revealed by reconstitution of SV40 DNA replication *in vitro*. Nature.

[17] Tsurimoto T., Stillman B. (1991). Replication factors required for SV40 DNA replication *in vitro*. II. Switching of DNA polymerase α and δ during initiation of leading and lagging strand synthesis. J. Biol. Chem..

[18] Tsurimoto T., Melendy T., Stillman B. (1990). Sequential initiation of lagging and leading strand synthesis by two different polymerase complexes at the SV40 DNA replication origin. Nature.

[19] Pursell Z.F., Isoz I., Lundström E.-B., Johansson E., Kunkel T.A. (2007). Yeast DNA polymerase ε participates in leading-strand DNA replication. Science.

[20] Nick McElhinny S.A., Gordenin D.A., Stith C.M., Burgers P.M., Kunkel T.A. (2008). Division of labor at the eukaryotic replication fork. Mol. Cell.

[21] Larrea A.A., Lujan S.A., Nick McElhinny S.A., Mieczkowski P.A., Resnick M.A., Gordenin D.A., Kunkel T.A. (2010). Genome-wide model for the normal eukaryotic DNA replication fork. Proc. Natl. Acad. Sci. USA.

[22] Neuwald A.F., Aravind L., Spouge J.L., Koonin E.V. (1999). AAA^+^: A class of chaperone-like ATPases associated with the assembly, operation, and disassembly of protein complexes. Genome Res..

[23] Labib K., Tercero J.A., Diffley J.F.X. (2000). Uninterrupted MCM2-7 function required for DNA replication fork progression. Science.

[24] Pacek M., Walter J.C. (2004). A requirement for MCM7 and Cdc45 in chromosome unwinding during eukaryotic DNA replication. EMBO J..

[25] Aparicio O.M., Weinstein D.M., Bell S.P. (1997). Components and Dynamics of DNA Replication Complexes in *S. cerevisiae*: Redistribution of MCM Proteins and Cdc45p during S Phase. Cell.

[26] Tye B.-K. (1994). The MCM2-3-5 proteins: Are they replication licensing factors?. Trends Cell Biol..

[27] Chong J.P.J., Hayashi M.K., Simon M.N., Xu R.-M., Stillman B. (2000). A double-hexamer archaeal minichromosome maintenance protein is an ATP-dependent DNA helicase. Proc. Natl. Acad. Sci. USA.

[28] Ishimi Y. (1997). A DNA helicase activity is associated with an MCM4, -6, and -7 protein complex. J. Biol. Chem..

[29] Lee J.K., Hurwitz J. (2000). Isolation and characterization of various complexes of the minichromosome maintenance proteins of *Schizosaccharomyces pombe*. J. Biol. Chem..

[30] Nishitani H., Lygerou Z. (2002). Control of DNA replication licensing in a cell cycle. Genes Cells.

[31] Lei M., Tye B. (2001). Initiating DNA synthesis: From recruiting to activating the MCM complex. J. Cell Sci..

[32] Méchali M. (2010). Eukaryotic DNA replication origins: Many choices for appropriate answers. Nat. Rev. Mol. Cell Biol..

[33] Coleman T.R., Carpenter P.B., Dunphy W.G. (1996). The *Xenopus* Cdc6 protein is essential for the initiation of a single round of DNA replication in cell-free extracts. Cell.

[34] Tanaka T., Knapp D., Nasmyth K. (1997). Loading of an Mcm protein onto DNA replication origins is regulated by Cdc6p and CDKs. Cell.

[35] Ogawa Y., Takahashi T., Masukata H. (1999). Association of fission yeast Orp1 and Mcm6 proteins with chromosomal replication origins. Mol. Cell. Biol..

[36] Maiorano D., Moreau J., Mechali M. (2000). XCDT1 is required for the assembly of pre-replicative complexes in *Xenopus laevis*. Nature.

[37] Nishitani H., Lygerou Z., Nishimoto T., Nurse P. (2000). The Cdt1 protein is required to license DNA for replication in fission yeast. Nature.

[38] Remus D., Beuron F., Tolun G., Griffith J.D., Morris E.P., Diffley J.F. (2009). Concerted loading of Mcm2-7 double hexamers around DNA during DNA replication origin licensing. Cell.

[39] Evrin C., Clarke P., Zech J., Lurz R., Sun J., Uhle S., Li H., Stillman B., Speck C. (2009). A double-hexameric MCM2-7 complex is loaded onto origin DNA during licensing of eukaryotic DNA replication. Proc. Natl. Acad. Sci. USA.

[40] Dahmann C., Diffley J.F., Nasmyth K.A. (1995). S-phase-promoting cyclin-dependent kinases prevent re-replication by inhibiting the transition of replication origins to a pre-replicative state. Curr. Biol..

[41] Mimura S., Takisawa H. (1998). *Xenopus* Cdc45-dependent loading of DNA polymerase α onto chromatin under the control of S-phase Cdk. EMBO J..

[42] Zou L., Stillman B. (1998). Formation of a preinitiation complex by S-phase cyclin CDK-dependent loading of Cdc45p onto chromatin. Science.

[43] Nougarede R., Della Seta F., Zarzov P., Schwob E. (2000). Hierarchy of S-phase-promoting factors: Yeast Dbf4-Cdc7 kinase requires prior S-phase cyclin-dependent kinase activation. Mol. Cell. Biol..

[44] Sheu Y.J., Stillman B. (2010). The Dbf4-Cdc7 kinase promotes S phase by alleviating an inhibitory activity in Mcm4. Nature.

[45] Zou L., Stillman B. (2000). Assembly of a complex containing Cdc45p, replication protein A, and Mcm2p at replication origins controlled by S-phase cyclin-dependent kinases and Cdc7p-Dbf4p kinase. Mol. Cell. Biol..

[46] Masai H., Taniyama C., Ogino K., Matsui E., Kakusho N., Matsumoto S., Kim J.M., Ishii A., Tanaka T., Kobayashi T. (2006). Phosphorylation of MCM4 by Cdc7 kinase facilitates its interaction with Cdc45 on the chromatin. J. Biol. Chem..

[47] Muramatsu S., Hirai K., Tak Y.-S., Kamimura Y., Araki H. (2010). CDK-dependent complex formation between replication proteins Dpb11, Sld2, Pol ε, and GINS in budding yeast. Genes Dev..

[48] Handa T., Kanke M., Takahashi T.S., Nakagawa T., Masukata H. (2012). DNA polymerization-independent functions of DNA polymerase epsilon in assembly and progression of the replisome in fission yeast. Mol. Biol. Cell.

[49] Saxena S., Dutta A. (2005). Geminin-Cdt1 balance is critical for genetic stability. Mutat. Res. Fundam. Mol. Mech. Mutagen..

[50] Mendez J., Zou-Yang X.H., Kim S.Y., Hidaka M., Tansey W.P., Stillman B. (2002). Human origin recognition complex large subunit is degraded by ubiquitin-mediated proteolysis after initiation of DNA replication. Mol. Cell.

[51] Diffley J.F., Cocker J.H., Dowell S.J., Rowley A. (1994). Two steps in the assembly of complexes at yeast replication origins* in vivo*. Cell.

[52] Liang C., Stillman B. (1997). Persistent initiation of DNA replication and chromatin-bound MCM proteins during the cell cycle in *cdc6* mutants. Genes Dev..

[53] Lee K.Y., Bang S.W., Yoon S.W., Lee S.-H., Yoon J.-B., Hwang D.S. (2012). Phosphorylation of ORC2 protein dissociates origin recognition complex from chromatin and replication origins. J. Biol. Chem..

[54] Saha P., Chen J., Thome K.C., Lawlis S.J., Hou Z.H., Hendricks M., Parvin J.D., Dutta A. (1998). Human CDC6/Cdc18 associates with Orc1 and cyclin-cdk and is selectively eliminated from the nucleus at the onset of S phase. Mol. Cell. Biol..

[55] Wohlschlegel J.A., Dwyer B.T., Dhar S.K., Cvetic C., Walter J.C., Dutta A. (2000). Inhibition of eukaryotic DNA replication by geminin binding to Cdt1. Science.

[56] Hook S.S., Lin J.J., Dutta A. (2007). Mechanisms to control rereplication and implications for cancer. Curr. Opin. Cell Biol..

[57] O'Connell B.C., Harper J.W. (2007). Ubiquitin proteasome system (UPS): What can chromatin do for you?. Curr. Opin. Cell Biol..

[58] Bravo R., Frank R., Blundell P.A., Macdonald-Bravo H. (1987). Cyclin/PCNA is the auxiliary protein of DNA polymerase-δ. Nature.

[59] Prelich G., Tan C.K., Kostura M., Mathews M.B., So A.G., Downey K.M., Stillman B. (1987). Functional identity of proliferating cell nuclear antigen and a DNA polymerase-δ auxiliary protein. Nature.

[60] Moldovan G.L., Pfander B., Jentsch S. (2007). PCNA, the maestro of the replication fork. Cell.

[61] Hoege C., Pfander B., Moldovan G.-L., Pyrowolakis G., Jentsch S. (2002). RAD6-dependent DNA repair is linked to modification of PCNA by ubiquitin and SUMO. Nature.

[62] Kannouche P.L., Wing J., Lehmann A.R. (2004). Interaction of human DNA polymerase η with monoubiquitinated PCNA: A possible mechanism for the polymerase switch in response to DNA damage. Mol. Cell.

[63] Yang X.H., Shiotani B., Classon M., Zou L. (2008). Chk1 and Claspin potentiate PCNA ubiquitination. Genes Dev..

[64] Branzei D., Seki M., Enomoto T. (2004). Rad18/Rad5/Mms2-mediated polyubiquitination of PCNA is implicated in replication completion during replication stress. Genes Cells.

[65] Blastyak A., Pinter L., Unk I., Prakash L., Prakash S., Haracska L. (2007). Yeast Rad5 protein required for postreplication repair has a DNA helicase activity specific for replication fork regression. Mol. Cell.

[66] Branzei D., Vanoli F., Foiani M. (2008). SUMOylation regulates Rad18-mediated template switch. Nature.

[67] Ulrich H.D., Vogel S., Davies A.A. (2005). SUMO keeps a check on recombination during DNA replication. Cell Cycle.

[68] Leach C.A., Michael W.M. (2005). Ubiquitin/SUMO modification of PCNA promotes replication fork progression in *Xenopus laevis* egg extracts. J. Cell Biol..

[69] Pfander B., Moldovan G.-L., Sacher M., Hoege C., Jentsch S. (2005). SUMO-modified PCNA recruits Srs2 to prevent recombination during S phase. Nature.

[70] Papouli E., Chen S., Davies A.A., Huttner D., Krejci L., Sung P., Ulrich H.D. (2005). Crosstalk between SUMO and ubiquitin on PCNA is mediated by recruitment of the helicase Srs2p. Mol. Cell.

[71] Parnas O., Zipin-Roitman A., Pfander B., Liefshitz B., Mazor Y., Ben-Aroya S., Jentsch S., Kupiec M. (2010). Elg1, an alternative subunit of the RFC clamp loader, preferentially interacts with SUMOylated PCNA. EMBO J..

[72] Naryzhny S.N., Lee H. (2004). The post-translational modifications of proliferating cell nuclear antigen: Acetylation, not phosphorylation, plays an important role in the regulation of its function. J. Biol. Chem..

[73] Cai J., Yao N., Gibbs E., Finkelstein J., Phillips B., O'Donnell M., Hurwitz J. (1998). ATP hydrolysis catalyzed by human replication factor C requires participation of multiple subunits. Proc. Natl. Acad. Sci. USA.

[74] Tsurimoto T., Stillman B. (1991). Replication factors required for SV40 DNA replication *in vitro*. I. DNA structure-specific recognition of a primer-template junction by eukaryotic DNA polymerases and their accessory proteins. J. Biol. Chem..

[75] Podust L.M., Podust V.N., Sogo J.M., Hubscher U. (1995). Mammalian DNA polymerase auxiliary proteins: Analysis of replication factor C-catalyzed proliferating cell nuclear antigen loading onto circular double-stranded DNA. Mol. Cell. Biol..

[76] Zhang G., Gibbs E., Kelman Z., O'Donnell M., Hurwitz J. (1999). Studies on the interactions between human replication factor C and human proliferating cell nuclear antigen. Proc. Natl. Acad. Sci. USA.

[77] Yao N.Y., Johnson A., Bowman G.D., Kuriyan J., O'Donnell M. (2006). Mechanism of proliferating cell nuclear antigen clamp opening by replication factor C. J. Biol. Chem..

[78] Skibbens R.V., Corson L.B., Koshland D., Hieter P. (1999). Ctf7p is essential for sister chromatid cohesion and links mitotic chromosome structure to the DNA replication machinery. Genes Dev..

[79] Lengronne A., McIntyre J., Katou Y., Kanoh Y., Hopfner K.P., Shirahige K., Uhlmann F. (2006). Establishment of sister chromatid cohesion at the *S. cerevisiae* replication fork. Mol. Cell.

[80] Ansbach A.B., Noguchi C., Klansek I.W., Heidlebaugh M., Nakamura T.M., Noguchi E. (2008). RFC^Ctf18^ and the Swi1-Swi3 complex function in separate and redundant pathways required for the stabilization of replication forks to facilitate sister chromatid cohesion in *Schizosaccharomyces pombe*. Mol. Biol. Cell.

[81] Terret M.E., Sherwood R., Rahman S., Qin J., Jallepalli P.V. (2009). Cohesin acetylation speeds the replication fork. Nature.

[82] Bermudez V.P., Lindsey-Boltz L.A., Cesare A.J., Maniwa Y., Griffith J.D., Hurwitz J., Sancar A. (2003). Loading of the human 9-1-1 checkpoint complex onto DNA by the checkpoint clamp loader hRad17-replication factor C complex *in vitro*. Proc. Natl. Acad. Sci. USA.

[83] Garg P., Stith C.M., Sabouri N., Johansson E., Burgers P.M. (2004). Idling by DNA polymerase δ maintains a ligatable nick during lagging-strand DNA replication. Genes Dev..

[84] Burgers P.M.J. (2009). Polymerase dynamics at the eukaryotic DNA replication fork. J. Biol. Chem..

[85] Jin Y.H., Ayyagari R., Resnick M.A., Gordenin D.A., Burgers P.M.J. (2003). Okazaki fragment maturation in yeast. J. Biol. Chem..

[86] Budd M.E., Campbell J.L. (1997). A yeast replicative helicase, Dna2 helicase, interacts with yeast FEN-1 nuclease in carrying out its essential function. Mol. Cell. Biol..

[87] Lee K.H., Kim D.W., Bae S.H., Kim J.A., Ryu G.H., Kwon Y.N., Kim K.A., Koo H.S., Seo Y.S. (2000). The endonuclease activity of the yeast Dna2 enzyme is essential *in vivo*. Nucleic Acids Res..

[88] Kao H.-I., Veeraraghavan J., Polaczek P., Campbell J.L., Bambara R.A. (2004). On the roles of *Saccharomyces cerevisiae* Dna2p and flap endonuclease 1 in Okazaki fragment processing. J. Biol. Chem..

[89] Sogo J.M., Lopes M., Foiani M. (2002). Fork reversal and ssDNA accumulation at stalled replication forks owing to checkpoint defects. Science.

[90] Byun T.S., Pacek M., Yee M.-C., Walter J.C., Cimprich K.A. (2005). Functional uncoupling of MCM helicase and DNA polymerase activities activates the ATR-dependent checkpoint. Genes Dev..

[91] Sheu Y.-J., Stillman B. (2006). Cdc7-Dbf4 Phosphorylates MCM Proteins via a Docking Site-Mediated Mechanism to Promote S Phase Progression. Mol. Cell.

[92] Matsumoto S., Shimmoto M., Kakusho N., Yokoyama M., Kanoh Y., Hayano M., Russell P., Masai H. (2010). Hsk1 kinase and Cdc45 regulate replication stress-induced checkpoint responses in fission yeast. Cell Cycle.

[93] Takayama Y., Kamimura Y., Okawa M., Muramatsu S., Sugino A., Araki H. (2003). GINS, a novel multiprotein complex required for chromosomal DNA replication in budding yeast. Genes Dev..

[94] Kubota Y., Takase Y., Komori Y., Hashimoto Y., Arata T., Kamimura Y., Araki H., Takisawa H. (2003). A novel ring-like complex of *Xenopus* proteins essential for the initiation of DNA replication. Genes Dev..

[95] Gambus A., Jones R.C., Sanchez-Diaz A., Kanemaki M., van Deursen F., Edmondson R.D., Labib K. (2006). GINS maintains association of Cdc45 with MCM in replisome progression complexes at eukaryotic DNA replication forks. Nat. Cell Biol..

[96] Moyer S.E., Lewis P.W., Botchan M.R. (2006). Isolation of the Cdc45/Mcm2-7/GINS (CMG) complex, a candidate for the eukaryotic DNA replication fork helicase. Proc. Natl. Acad. Sci. USA.

[97] Im J.S., Ki S.H., Farina A., Jung D.S., Hurwitz J., Lee J.K. (2009). Assembly of the Cdc45-Mcm2-7-GINS complex in human cells requires the Ctf4/And-1, RecQL4, and Mcm10 proteins. Proc. Natl. Acad. Sci. USA.

[98] Ilves I., Petojevic T., Pesavento J.J., Botchan M.R. (2010). Activation of the MCM2-7 helicase by association with Cdc45 and GINS proteins. Mol. Cell.

[99] Costa A., Ilves I., Tamberg N., Petojevic T., Nogales E., Botchan M.R., Berger J.M. (2011). The structural basis for MCM2-7 helicase activation by GINS and Cdc45. Nat. Struct. Mol. Biol..

[100] Matsuoka S., Ballif B.A., Smogorzewska A., McDonald E.R., Hurov K.E., Luo J., Bakalarski C.E., Zhao Z., Solimini N., Lerenthal Y. (2007). ATM and ATR substrate analysis reveals extensive protein networks responsive to DNA damage. Science.

[101] Bermudez V.P., Farina A., Tappin I., Hurwitz J. (2009). Influence of the human cohesion establishment factor Ctf4/AND-1 on DNA replication. J. Biol. Chem..

[102] Gambus A., van Deursen F., Polychronopoulos D., Foltman M., Jones R.C., Edmondson R.D., Calzada A., Labib K. (2009). A key role for Ctf4 in coupling the MCM2-7 helicase to DNA polymerase α within the eukaryotic replisome. EMBO J..

[103] Zhu W., Ukomadu C., Jha S., Senga T., Dhar S.K., Wohlschlegel J.A., Nutt L.K., Kornbluth S., Dutta A. (2007). Mcm10 and And-1/CTF4 recruit DNA polymerase α to chromatin for initiation of DNA replication. Genes Dev..

[104] Lou H., Komata M., Katou Y., Guan Z., Reis C.C., Budd M., Shirahige K., Campbell J.L. (2008). Mrc1 and DNA polymerase ε function together in linking DNA replication and the S phase checkpoint. Mol. Cell.

[105] Petermann E., Helleday T., Caldecott K.W. (2008). Claspin promotes normal replication fork rates in human cells. Mol. Biol. Cell.

[106] Leman A.R., Noguchi E. (2012). Local and global functions of Timeless and Tipin in replication fork protection. Cell Cycle.

[107] Tourriere H., Versini G., Cordon-Preciado V., Alabert C., Pasero P. (2005). Mrc1 and Tof1 promote replication fork progression and recovery independently of Rad53. Mol. Cell.

[108] Unsal-Kacmaz K., Chastain P.D., Qu P.-P., Minoo P., Cordeiro-Stone M., Sancar A., Kaufmann W.K. (2007). The human Tim/Tipin complex coordinates an intra-S checkpoint response to UV that slows replication fork displacement. Mol. Cell. Biol..

[109] Brown E.J., Baltimore D. (2000). ATR disruption leads to chromosomal fragmentation and early embryonic lethality. Genes Dev..

[110] De Klein A., Muijtjens M., van Os R., Verhoeven Y., Smit B., Carr A.M., Lehmann A.R., Hoeijmakers J.H. (2000). Targeted disruption of the cell-cycle checkpoint gene ATR leads to early embryonic lethality in mice. Curr. Biol..

[111] Cortez D., Guntuku S., Qin J., Elledge S.J. (2001). ATR and ATRIP: Partners in checkpoint signaling. Science.

[112] Zou L., Elledge S.J. (2003). Sensing DNA damage through ATRIP recognition of RPA-ssDNA complexes. Science.

[113] Dart D.A., Adams K.E., Akerman I., Lakin N.D. (2004). Recruitment of the cell cycle checkpoint kinase ATR to chromatin during S-phase. J. Biol. Chem..

[114] Cha R.S., Kleckner N. (2002). ATR homolog Mec1 promotes fork progression, thus averting breaks in replication slow zones. Science.

[115] Ball H.L., Myers J.S., Cortez D. (2005). ATRIP binding to replication protein A-single-stranded DNA promotes ATR-ATRIP localization but is dispensable for Chk1 phosphorylation. Mol. Biol. Cell.

[116] Zou L., Cortez D., Elledge S.J. (2002). Regulation of ATR substrate selection by Rad17-dependent loading of Rad9 complexes onto chromatin. Genes Dev..

[117] Parrilla-Castellar E.R., Karnitz L.M. (2003). Cut5 is required for the binding of Atr and DNA polymerase α to genotoxin-damaged chromatin. J. Biol. Chem..

[118] Kumagai A., Lee J., Yoo H.Y., Dunphy W.G. (2006). TopBP1 Activates the ATR-ATRIP Complex. Cell.

[119] Lee J., Kumagai A., Dunphy W.G. (2007). The Rad9-Hus1-Rad1 checkpoint clamp regulates interaction of TopBP1 with ATR. J. Biol. Chem..

[120] Yan S., Michael W.M. (2009). TopBP1 and DNA polymerase-α directly recruit the 9-1-1 complex to stalled DNA replication forks. J. Cell Biol..

[121] Kumagai A., Dunphy W.G. (2000). Claspin, a Novel Protein Required for the Activation of Chk1 during a DNA Replication Checkpoint Response in *Xenopus* Egg Extracts. Mol. Cell.

[122] Chini C.C., Chen J. (2003). Human claspin is required for replication checkpoint control. J. Biol. Chem..

[123] Kumagai A., Kim S.-M., Dunphy W.G. (2004). Claspin and the activated form of ATR-ATRIP collaborate in the activation of Chk1. J. Biol. Chem..

[124] Kumagai A., Dunphy W.G. (2003). Repeated phosphopeptide motifs in Claspin mediate the regulated binding of Chk1. Nat. Cell Biol..

[125] Jin J., Shirogane T., Xu L., Nalepa G., Qin J., Elledge S.J., Harper J.W. (2003). SCF-ßTRCP links Chk1 signaling to degradation of the Cdc25A protein phosphatase. Genes Dev..

[126] Sanchez Y., Wong C., Thoma R.S., Richman R., Wu Z., Piwnica-Worms H., Elledge S.J. (1997). Conservation of the Chk1 checkpoint pathway in mammals: Linkage of DNA damage to Cdk regulation through Cdc25. Science.

[127] Peng C.-Y., Graves P.R., Thoma R.S., Wu Z., Shaw A.S., Piwnica-Worms H. (1997). Mitotic and G2 checkpoint control: Regulation of 14-3-3 protein binding by phosphorylation of Cdc25C on serine-216. Science.

[128] Kumagai A., Guo Z., Emami K.H., Wang S.X., Dunphy W.G. (1998). The *Xenopus* Chk1 protein kinase mediates a caffeine-sensitive pathway of checkpoint control in cell-free extracts. J. Cell Biol..

[129] Miao H., Seiler J.A., Burhans W.C. (2003). Regulation of cellular and SV40 virus origins of replication by Chk1-dependent intrinsic and UVC radiation-induced checkpoints. J. Biol. Chem..

[130] Sorensen C.S., Syljuasen R.G., Lukas J., Bartek J. (2004). ATR, Claspin and the Rad9-Rad1-Hus1 complex regulate Chk1 and Cdc25A in the absence of DNA damage. Cell Cycle.

[131] Syljuasen R.G., Sorensen C.S., Hansen L.T., Fugger K., Lundin C., Johansson F., Helleday T., Sehested M., Lukas J., Bartek J. (2005). Inhibition of human Chk1 causes increased initiation of DNA replication, phosphorylation of ATR targets, and DNA breakage. Mol. Cell. Biol..

[132] Kornberg R.D. (1974). Chromatin structure: A repeating unit of histones and DNA. Science.

[133] Kornberg R.D., Thomas J.O. (1974). Chromatin structure; oligomers of the histones. Science.

[134] Benyajati C., Worcel A. (1976). Isolation, characterization, and structure of the folded interphase genome of *Drosophila melanogaste*. Cell.

[135] Luger K., Mader A.W., Richmond R.K., Sargent D.F., Richmond T.J. (1997). Crystal structure of the nucleosome core particle at 2.8Å resolution. Nature.

[136] Reinberg D., Sims R.J. (2006). de FACTo nucleosome dynamics. J. Biol. Chem..

[137] Wittmeyer J., Joss L., Formosa T. (1999). Spt16 and Pob3 of *Saccharomyces cerevisiae* form an essential, abundant heterodimer that is nuclear, chromatin-associated, and copurifies with DNA polymerase α. Biochemistry.

[138] Wittmeyer J., Formosa T. (1997). The *Saccharomyces cerevisiae* DNA polymerase alpha catalytic subunit interacts with Cdc68/Spt16 and with Pob3, a protein similar to an HMG1-like protein. Mol. Cell. Biol..

[139] Formosa T., Eriksson P., Wittmeyer J., Ginn J., Yu Y., Stillman D.J. (2001). Spt16-Pob3 and the HMG protein Nhp6 combine to form the nucleosome-binding factor SPN. EMBO J..

[140] Tan B.C., Chien C.T., Hirose S., Lee S.C. (2006). Functional cooperation between FACT and MCM helicase facilitates initiation of chromatin DNA replication. EMBO J..

[141] Tan B.C., Liu H., Lin C.L., Lee S.C. (2010). Functional cooperation between FACT and MCM is coordinated with cell cycle and differential complex formation. J. Biomed. Sci..

[142] Han J., Li Q., McCullough L., Kettelkamp C., Formosa T., Zhang Z. (2010). Ubiquitylation of FACT by the cullin-E3 ligase Rtt101 connects FACT to DNA replication. Genes Dev..

[143] Xin H., Takahata S., Blanksma M., McCullough L., Stillman D.J., Formosa T. (2009). yFACT induces global accessibility of nucleosomal DNA without H2A-H2B displacement. Mol. Cell.

[144] Groth A., Corpet A., Cook A.J., Roche D., Bartek J., Lukas J., Almouzni G. (2007). Regulation of replication fork progression through histone supply and demand. Science.

[145] Mello J.A., Sillje H.H., Roche D.M., Kirschner D.B., Nigg E.A., Almouzni G. (2002). Human Asf1 and CAF-1 interact and synergize in a repair-coupled nucleosome assembly pathway. EMBO Rep..

[146] Daganzo S.M., Erzberger J.P., Lam W.M., Skordalakes E., Zhang R., Franco A.A., Brill S.J., Adams P.D., Berger J.M., Kaufman P.D. (2003). Structure and function of the conserved core of histone deposition protein Asf1. Curr. Biol..

[147] Groth A., Ray-Gallet D., Quivy J.P., Lukas J., Bartek J., Almouzni G. (2005). Human Asf1 regulates the flow of S phase histones during replicational stress. Mol. Cell.

[148] Stillman B. (1986). Chromatin assembly during SV40 DNA replication *in vitro*. Cell.

[149] Smith S., Stillman B. (1989). Purification and characterization of CAF-I, a human cell factor required for chromatin assembly during DNA replication *in vitro*. Cell.

[150] Verreault A., Kaufman P.D., Kobayashi R., Stillman B. (1996). Nucleosome assembly by a complex of CAF-1 and acetylated histones H3/H4. Cell.

[151] Shibahara K., Stillman B. (1999). Replication-dependent marking of DNA by PCNA facilitates CAF-1-coupled inheritance of chromatin. Cell.

[152] Rolef Ben-Shahar T., Castillo A.G., Osborne M.J., Borden K.L., Kornblatt J., Verreault A. (2009). Two fundamentally distinct PCNA interaction peptides contribute to chromatin assembly factor 1 function. Mol. Cell. Biol..

[153] Huang S., Zhou H., Katzmann D., Hochstrasser M., Atanasova E., Zhang Z. (2005). Rtt106p is a histone chaperone involved in heterochromatin-mediated silencing. Proc. Natl. Acad. Sci. USA.

[154] Huang S., Zhou H., Tarara J., Zhang Z. (2007). A novel role for histone chaperones CAF-1 and Rtt106p in heterochromatin silencing. EMBO J..

[155] Ransom M., Dennehey B.K., Tyler J.K. (2010). Chaperoning histones during DNA replication and repair. Cell.

[156] Ito T., Tyler J.K., Bulger M., Kobayashi R., Kadonaga J.T. (1996). ATP-facilitated chromatin assembly with a nucleoplasmin-like protein from *Drosophila melanogaster*. J. Biol. Chem..

[157] Gasser R., Koller T., Sogo J.M. (1996). The stability of nucleosomes at the replication fork. J. Mol. Biol..

[158] Rothstein R., Michel B.N.D., Gangloff S. (2000). Replication fork pausing and recombination or “gimme a break”. Genes Dev..

[159] Bastia D., Zzaman S., Krings G., Saxena M., Peng X., Greenberg M.M. (2008). Replication termination mechanism as revealed by Tus-mediated polar arrest of a sliding helicase. Proc. Natl. Acad. Sci. USA.

[160] Brewer B.J., Fangman W.L. (1988). A replication fork barrier at the 3' end of yeast ribosomal RNA genes. Cell.

[161] Linskens M.H., Huberman J.A. (1988). Organization of replication of ribosomal DNA in *Saccharomyces cerevisiae*. Mol. Cell. Biol..

[162] Kobayashi T., Horiuchi T. (1996). A yeast gene product, Fob1 protein, required for both replication fork blocking and recombinational hotspot activities. Genes Cells.

[163] Kobayashi T. (2003). The replication fork barrier site forms a unique structure with Fob1p and inhibits the replication fork. Mol. Cell. Biol..

[164] Johzuka K., Horiuchi T. (2002). Replication fork block protein, Fob1, acts as an rDNA region specific recombinator in *S. cerevisiae*. Genes Cells.

[165] Mohanty B.K., Bairwa N.K., Bastia D. (2006). The Tof1p-Csm3p protein complex counteracts the Rrm3p helicase to control replication termination of *Saccharomyces cerevisiae*. Proc. Natl. Acad. Sci. USA.

[166] Ivessa A.S., Zhou J.Q., Zakian V.A. (2000). The *Saccharomyces* Pif1p DNA helicase and the highly related Rrm3p have opposite effects on replication fork progression in ribosomal DNA. Cell.

[167] Ivessa A.S., Zhou J.Q., Schulz V.P., Monson E.K., Zakian V.A. (2002). *Saccharomyces* Rrm3p, a 5' to 3' DNA helicase that promotes replication fork progression through telomeric and subtelomeric DNA. Genes Dev..

[168] Ivessa A.S., Lenzmeier B.A., Bessler J.B., Goudsouzian L.K., Schnakenberg S.L., Zakian V.A. (2003). The *Saccharomyces cerevisiae* helicase Rrm3p facilitates replication past nonhistone protein-DNA complexes. Mol. Cell.

[169] Torres J.Z., Schnakenberg S.L., Zakian V.A. (2004). *Saccharomyces cerevisiae* Rrm3p DNA helicase promotes genome integrity by preventing replication fork stalling: Viability of rrm3 cells requires the intra-S-phase checkpoint and fork restart activities. Mol. Cell. Biol..

[170] Torres J.Z., Bessler J.B., Zakian V.A. (2004). Local chromatin structure at the ribosomal DNA causes replication fork pausing and genome instability in the absence of the *S. cerevisiae* DNA helicase Rrm3p. Genes Dev..

[171] Schmidt K.H., Derry K.L., Kolodner R.D. (2002). *Saccharomyces cerevisiae* RRM3, a 5' to 3' DNA helicase, physically interacts with proliferating cell nuclear antigen. J. Biol. Chem..

[172] Azvolinsky A., Dunaway S., Torres J.Z., Bessler J.B., Zakian V.A. (2006). The *S. cerevisiae* Rrm3p DNA helicase moves with the replication fork and affects replication of all yeast chromosomes. Genes Dev..

[173] Sanchez-Gorostiaga A., Lopez-Estrano C., Krimer D.B., Schvartzman J.B., Hernandez P. (2004). Transcription termination factor reb1p causes two replication fork barriers at its cognate sites in fission yeast ribosomal DNA *in vivo*. Mol. Cell. Biol..

[174] Krings G., Bastia D. (2004). *swi1*- and *swi3*-dependent and independent replication fork arrest at the ribosomal DNA of *Schizosaccharomyces pombe*. Proc. Natl. Acad. Sci. USA.

[175] Krings G., Bastia D. (2005). Sap1p binds to Ter1 at the ribosomal DNA of *Schizosaccharomyces pombe* and causes polar replication fork arrest. J. Biol. Chem..

[176] Mejia-Ramirez E., Sanchez-Gorostiaga A., Krimer D.B., Schvartzman J.B., Hernandez P. (2005). The mating type switch-activating protein Sap1 is required for replication fork arrest at the rRNA genes of fission yeast. Mol. Cell. Biol..

[177] Little R.D., Platt T.H., Schildkraut C.L. (1993). Initiation and termination of DNA replication in human rRNA genes. Mol. Cell. Biol..

[178] Evers R., Grummt I. (1995). Molecular coevolution of mammalian ribosomal gene terminator sequences and the transcription termination factor TTF-I. Proc. Natl. Acad. Sci. USA.

[179] Kanei-Ishii C., Sarai A., Sawazaki T., Nakagoshi H., He D.N., Ogata K., Nishimura Y., Ishii S. (1990). The tryptophan cluster: A hypothetical structure of the DNA-binding domain of the myb protooncogene product. J. Biol. Chem..

[180] Beach D.H. (1983). Cell type switching by DNA transposition in fission yeast. Nature.

[181] Beach D.H., Klar A.J. (1984). Rearrangements of the transposable mating-type cassettes of fission yeast. EMBO J..

[182] Dalgaard J.Z., Klar A.J. (1999). Orientation of DNA replication establishes mating-type switching pattern in *S. pombe*. Nature.

[183] Arcangioli B., de Lahondes R. (2000). Fission yeast switches mating type by a replication-recombination coupled process. EMBO J..

[184] Dalgaard J.Z., Klar A.J.S. (2001). A DNA replication-arrest site RTS1 regulates imprinting by determining the direction of replication at *mat1* in *S. pombe*. Genes Dev..

[185] Vengrova S., Codlin S., Dalgaard J.Z. (2002). RTS1-an eukaryotic terminator of replication. Int. J. Biochem. Cell Biol..

[186] Lambert S., Watson A., Sheedy D.M., Martin B., Carr A.M. (2005). Gross chromosomal rearrangements and elevated recombination at an inducible site-specific replication fork barrier. Cell.

[187] Ahn J.S., Osman F., Whitby M.C. (2005). Replication fork blockage by RTS1 at an ectopic site promotes recombination in fission yeast. EMBO J..

[188] Codlin S., Dalgaard J.Z. (2003). Complex mechanism of site-specific DNA replication termination in fission yeast. EMBO J..

[189] Eydmann T., Sommariva E., Inagawa T., Mian S., Klar A.J.S., Dalgaard J.Z. (2008). Rtf1-mediated eukaryotic site-specific replication termination. Genetics.

[190] Egel R., Beach D.H., Klar A.J. (1984). Genes required for initiation and resolution steps of mating-type switching in fission yeast. Proc. Natl. Acad. Sci. USA.

[191] Dalgaard J.Z., Klar A.J. (2000). *swi1* and *swi3* perform imprinting, pausing, and termination of DNA replication in *S. pombe*. Cell.

[192] Desany B.A., Alcasabas A.A., Bachant J.B., Elledge S.J. (1998). Recovery from DNA replicational stress is the essential function of the S-phase checkpoint pathway. Genes Dev..

[193] Zhao X., Muller E.G., Rothstein R. (1998). A suppressor of two essential checkpoint genes identifies a novel protein that negatively affects dNTP pools. Mol. Cell.

[194] Hashash N., Johnson A.L., Cha R.S. (2010). Regulation of fragile sites expression in budding yeast by MEC1, RRM3 and hydroxyurea. J. Cell Sci..

[195] Lopes M., Cotta-Ramusino C., Pellicioli A., Liberi G., Plevani P., Muzi-Falconi M., Newlon C.S., Foiani M. (2001). The DNA replication checkpoint response stabilizes stalled replication forks. Nature.

[196] Tercero J.A., Diffley J.F.X. (2001). Regulation of DNA replication fork progression through damaged DNA by the Mec1/Rad53 checkpoint. Nature.

[197] Magenis R.E., Hecht F., Lovrien E.W. (1970). Heritable fragile site on chromosome 16: Probable localization of Haptoglobin locus in man. Science.

[198] Harvey J., Judge C., Wiener S. (1977). Familial X-linked mental retardation with an X chromosome abnormality. J. Med. Genet..

[199] Sutherland G.R. (1977). Fragile sites on human chromosomes: Demonstration of their dependence on the type of tissue culture medium. Science.

[200] Sutherland G.R. (1979). Heritable fragile sites on human chromosomes I. Factors affecting expression in lymphocyte culture. Am. J. Hum. Genet..

[201] Glover T.W. (1981). FUdR induction of the X chromosome fragile site: Evidence for the mechanism of folic acid and thymidine inhibition. Am. J. Hum. Genet..

[202] Verkerk A.J., Pieretti M., Sutcliffe J.S., Fu Y.H., Kuhl D.P., Pizzuti A., Reiner O., Richards S., Victoria M.F., Zhang F.P. (1991). Identification of a gene (FMR-1) containing a CGG repeat coincident with a breakpoint cluster region exhibiting length variation in fragile X syndrome. Cell.

[203] Fu Y.H., Kuhl D.P., Pizzuti A., Pieretti M., Sutcliffe J.S., Richards S., Verkerk A.J., Holden J.J., Fenwick R.G., Warren S.T. (1991). Variation of the CGG repeat at the fragile X site results in genetic instability: Resolution of the Sherman paradox. Cell.

[204] Oberle I., Rousseau F., Heitz D., Kretz C., Devys D., Hanauer A., Boue J., Bertheas M.F., Mandel J.L. (1991). Instability of a 550-base pair DNA segment and abnormal methylation in fragile X syndrome. Science.

[205] Durkin S.G., Glover T.W. (2007). Chromosome fragile sites. Annu. Rev. Genet..

[206] Glover T.W., Berger C., Coyle J., Echo B. (1984). DNA polymerase α inhibition by aphidicolin induces gaps and breaks at common fragile sites in human chromosomes. Hum. Genet..

[207] Sutherland G.R., Parslow M.I., Baker E. (1985). New classes of common fragile sites induced by 5-azacytidine and bromodeoxyuridine. Hum. Genet..

[208] LeBeau M.M., Rowley J.D. (1984). Heritable fragile sites in cancer. Nature.

[209] Gacy A.M., Goellner G., Juranic N., Macura S., McMurray C.T. (1995). Trinucleotide repeats that expand in human disease form hairpin structures *in vitro*. Cell.

[210] Hewett D.R., Handt O., Hobson L., Mangelsdorf M., Eyre H.J., Baker E., Sutherland G.R., Schuffenhauer S., Mao J.I., Richards R.I. (1998). FRA10B structure reveals common elements in repeat expansion and chromosomal fragile site genesis. Mol. Cell.

[211] Zlotorynski E., Rahat A., Skaug J., Ben-Porat N., Ozeri E., Hershberg R., Levi A., Scherer S.W., Margalit H., Kerem B. (2003). Molecular basis for expression of common and rare fragile sites. Mol. Cell. Biol..

[212] Durkin S.G., Ragland R.L., Arlt M.F., Mulle J.G., Warren S.T., Glover T.W. (2008). Replication stress induces tumor-like microdeletions in FHIT/FRA3B. Proc. Natl. Acad. Sci. USA.

[213] Casper A.M., Nghiem P., Arlt M.F., Glover T.W. (2002). ATR regulates fragile site stability. Cell.

